# Supplementary material for: Drugging the Undruggable Trypanosoma brucei Monothiol Glutaredoxin 1
Source: Molecules. 2023 Jan 28;28(3):1276. doi: 10.3390/molecules28031276 (PMC9919793; doi:10.3390/molecules28031276)
Supplement: Supplementary file 1 [file molecules-28-01276-s001.zip › molecules-2109880-supplementary.pdf]

Drugging the undruggable Trypanosoma Brucei 1CGrx1

Annagiulia Favaro [1,†], Giovanni Bolcato [1,†], Marcelo A. Comini [2], Stefano Moro [1], Massimo Bellanda [3,4] and Mattia Sturlese [1,*]

[1] Molecular Modeling Section (MMS), Department of Pharmaceutical and Pharmacological Sciences, University of Padova, via Marzolo 5, Padova 35131, Italy
[2] Laboratory Redox Biology of Trypanosomes, Institut Pasteur de Montevideo, Mataojo 2020, 11400 Montevideo, Uruguay
[3] Department of Chemical Sciences, University of Padova, via Marzolo 1, Padova 35131, Italy
4 Consiglio Nazionale delle Ricerche, Institute of Biomolecular Chemistry, Padova 35131, Italy
* Correspondence: mattia.sturlese@unipd.it; Tel. +39-049-8275081
† These authors contributed equally to this work.

Supplementary Information

Figure S1. Overview of the calculated properties of the fragment library. All the descriptors were calculated by using MOE2020.

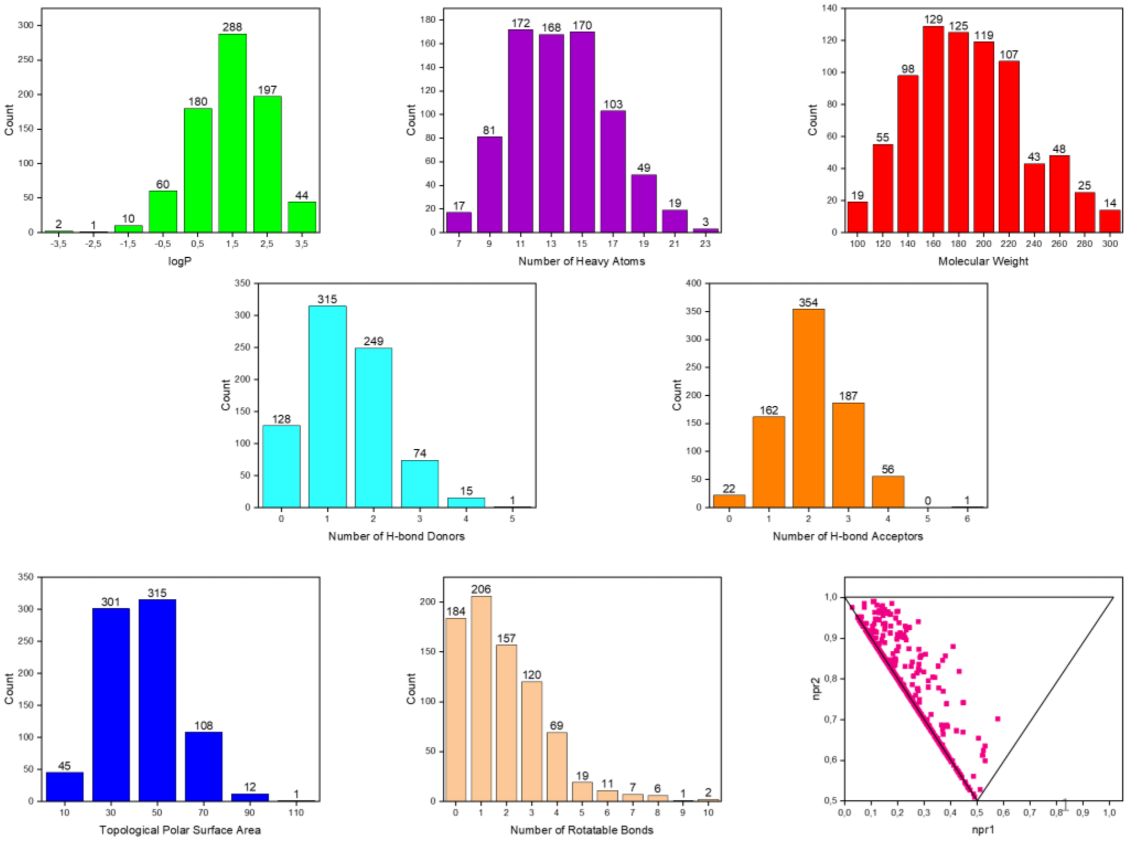

Figure S2. Alignment and comparison of the sequence for Grx1 (Trypanosoma Brucei) and Grx4 (Escherichia Coli). The residues that are close to the co-enzyme glutathione are circled in red. As it can be observed, the site is well conserved between the two.

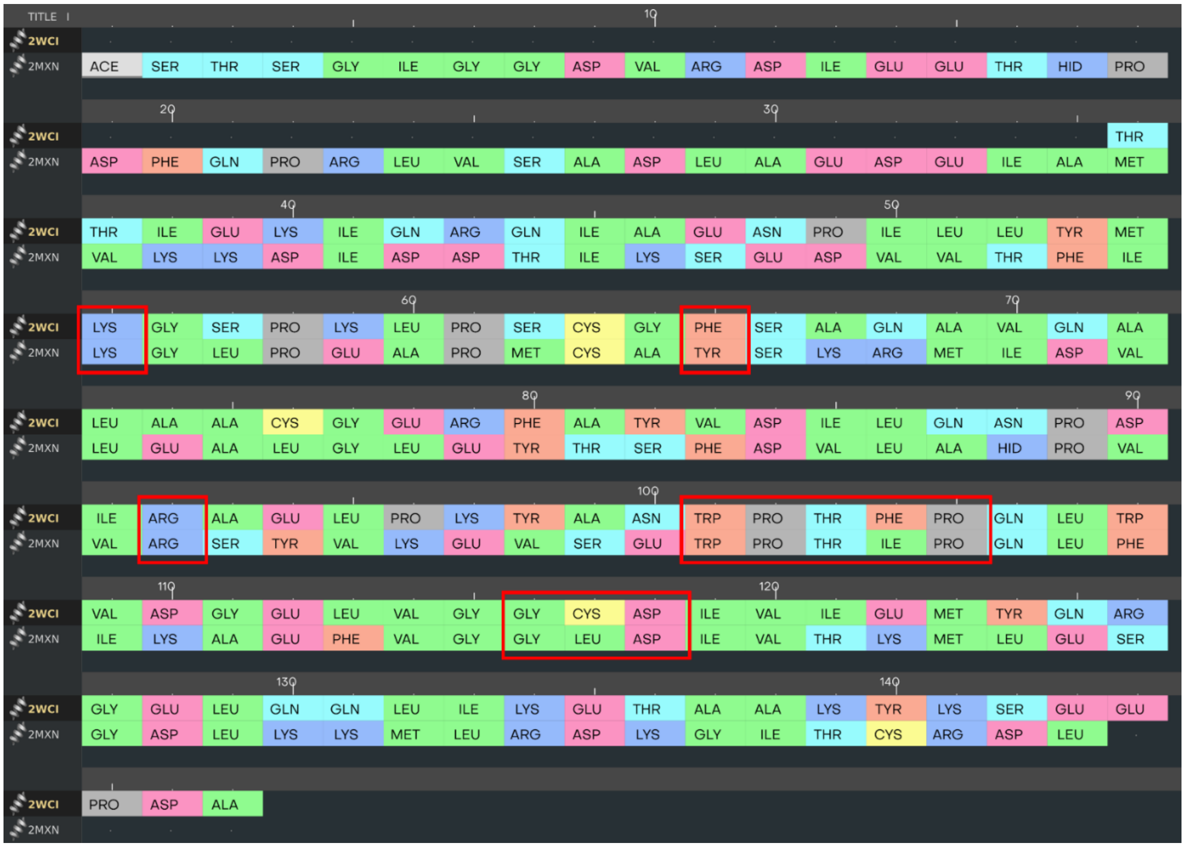

Figure S3. List of the compounds originated from VS.

1<br>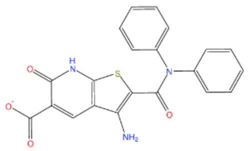<br>MolPort-002-404.4260<br>Weight: 404.4260	2<br>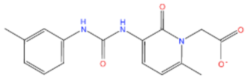<br>MolPort-035-314.3210<br>Weight: 314.3210	3<br>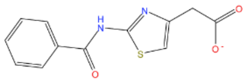<br>MolPort-002-261.2810<br>Weight: 261.2810	4<br>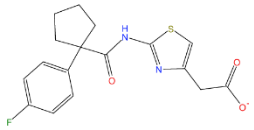<br>MolPort-005-347.3900<br>Weight: 347.3900
5<br>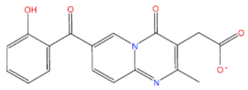<br>MolPort-009-337.3110<br>Weight: 337.3110	6<br>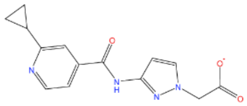<br>MolPort-046-285.2830<br>Weight: 285.2830	7<br>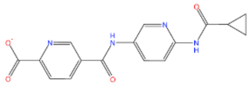<br>MolPort-047-325.3040<br>Weight: 325.3040	8<br>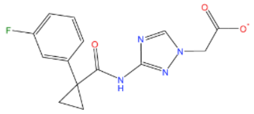<br>MolPort-045-303.2730<br>Weight: 303.2730
